# *Notes from the Field:* Dengue Outbreak — American Samoa, 2025–2026

**DOI:** 10.15585/mmwr.mm7544a2

**Published:** 2026-11-12

**Authors:** Emily Trautner, Adam Konrote, Romson Nuake, Aileen Solaita, Angelynn Papu, Sarona Toalepai, Elia Titiimaea, Annette Ilimaleota, Limb K. Hapairai, Mark Schmaedick, Dania M. Rodriguez, Yaritbel Torres-Mendoza, Janice Perez-Padilla, Ryan R. Hemme, Gilberto A. Santiago, Francisco S. Palomeque, Laura E. Adams, Gabriela Paz-Bailey, Forrest K. Jones

**Affiliations:** ^1^Division of Vector-Borne Diseases, National Center for Emerging and Zoonotic Infectious Diseases, CDC; ^2^American Samoa Department of Health, Tafuna, American Samoa; ^3^American Samoa Department of Health, Public Health Laboratory, Tafuna, American Samoa; ^4^Lyndon B. Johnson Hospital, Pago Pago, American Samoa; ^5^Pacific Island Health Officers’ Association, Honolulu, Hawaii; ^6^American Samoa Community College, Pago Pago, American Samoa;^ 7^Division of State and Local Readiness, Office of Readiness and Response, CDC.

**Summary**

**What is already known about this topic?**

Before July 2025, the last dengue outbreak in American Samoa occurred during 2016–2018, which prompted implementation of confirmatory dengue testing on the island.

**What is added by this report?**

A dengue outbreak was declared in American Samoa in July 2025. During June 2025–August 2026, the outbreak resulted in 1,048 confirmed cases and 371 (35%) hospitalizations; no deaths occurred. The majority of cases occurred among patients aged <20 years, and dengue virus 1 was the predominant serotype. Interventions focused on clinical preparedness and vector control, which included insecticide spraying and installation of spatial emanators.

**What are the implications for public health practice?**

This was the first dengue outbreak in American Samoa that had the benefit of on-island confirmatory testing, which facilitated timely identification and guided the outbreak response.

Dengue is a febrile illness caused by four distinct dengue viruses (DENV-1, DENV-2, DENV-3, and DENV-4). The virus is transmitted by infected *Aedes* mosquitoes (*1*). In 2025, several countries and territories in the Western Pacific Region experienced dengue outbreaks (*2*), and on June 20, 2025, American Samoa, a territory of the United States located in the south Pacific Ocean, reported its first positive dengue test result in 2025. Two weeks later, on July 8, American Samoa declared a public health emergency after identifying six dengue cases, including two locally acquired cases and four associated with travel to neighboring Samoa, where a large dengue outbreak was ongoing (*3*,*4*). Recent dengue outbreaks in American Samoa were associated with DENV-4 (2009), DENV-3 (2015), and DENV-2 (2016–2018) (*5*). After the 2016–2018 outbreak, American Samoa implemented confirmatory laboratory dengue testing on the island.

## Investigation and Outcomes

### Case Definitions

Suspected dengue cases, defined as the presence of fever plus one or more additional manifestation,* were reported to the American Samoa Department of Health (ASDOH) for confirmatory testing and travel history assessment. Patients with confirmed dengue cases had dengue like symptoms and positive blood test results by one or more of the following tests: 1) nonstructural protein 1 (NS1) antigen enzyme-linked immunosorbent assay (ELISA), 2) immunoglobulin M (IgM) ELISA, 3) reverse transcription–polymerase chain reaction (RT-PCR), or 4) a rapid dengue test^†^ at the ASDOH Public Health Laboratory (*1*). A subset of confirmed cases underwent DENV serotyping by RT-PCR testing at the State of Hawaii, Department of Health, Public Health & Environmental Laboratories.^§^ Patients with travel-associated cases were those who reported travel outside of American Samoa during the 14 days preceding symptom onset; cases in patients with no recent travel were classified as locally acquired. This outbreak investigation was reviewed by CDC, deemed not research, and conducted consistent with applicable federal law and CDC policy.^¶^

### Characteristics of Patients with Dengue

During June 2025–August 2026, a total of 1,048 confirmed dengue cases were identified (incidence = 211 cases per 10,000 population**). The majority of cases occurred among persons aged <20 years (814; 78%) and among males (650; 62%) (Supplementary Table). Incidence was highest among children aged 10–14 years (652 per 10,000). Overall, 49 (5%) cases were classified as travel associated, and 999 (95%) were locally acquired. A total of 371 (35%) patients required hospitalization; none died. The weekly number of hospital admissions peaked in December 2025 at 19 (median = 6; IQR = 4–9). On the day of highest occupancy, 18 of 110 (16%) available hospital beds^††^ were occupied by patients with dengue. The median length of hospitalization was 4 days (IQR = 3–5 days). Among all cases, 99% were confirmed using NS1 ELISA (838; 80%) or RT-PCR testing (204; 19%). Two cases were confirmed by IgM ELISA only and four by rapid test only. Among 146 serotyped cases, DENV-1 was identified in 136 (93%) and DENV-2 in 10 (7%).

### Temporal and Geographic Distributions of Cases

Case counts increased during June–August 2025; among 97 of these early cases, 38 (39%) were travel associated (Figure); however, during September 2025–February 2026, travel-associated cases accounted for 1% of all cases (11 of 783), consistent with sustained local transmission. No travel-associated cases were identified after February 15, 2026, and case counts consistently declined during February–August. Among American Samoa’s 76 villages, 54 (71%) reported at least one dengue case, with the highest number (152; 15%) reported in Tafuna, the most populous village on the island.

**FIGURE:**
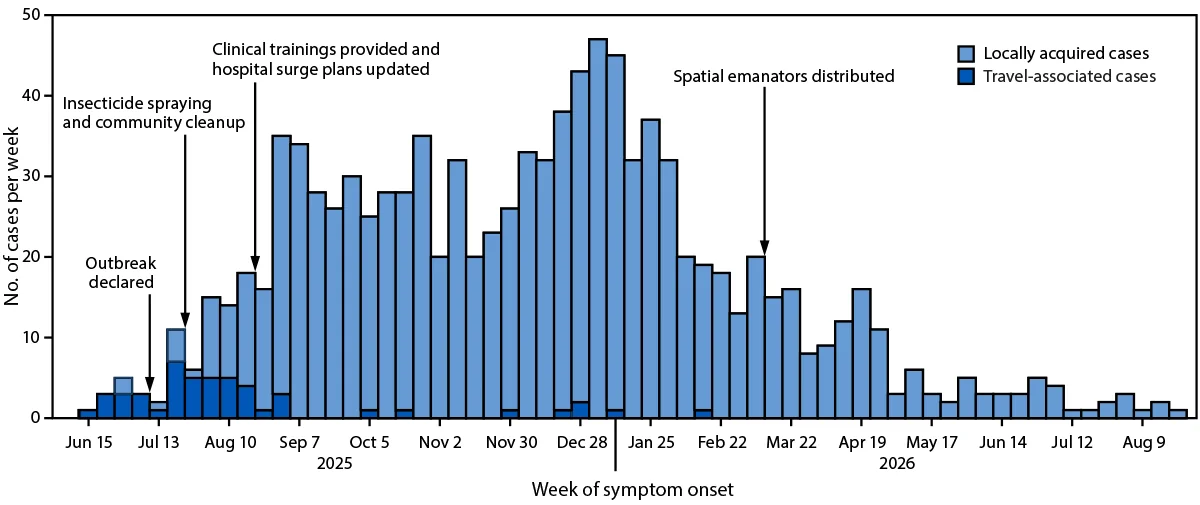
Number of travel-associated and locally acquired dengue cases, by week* of symptom onset and start date of public health interventions^†^ — American Samoa, June 2025–August 2026 * Weeks begin on Sunday and end on Saturday. ^†^ Spatial emanators are devices that slowly release a fast-acting insecticide into the air, without using heat or electricity, that reduces mosquitoes’ ability to bite. The spatial emanator used during the outbreak contained transfluthrin and had a stated use duration of 1 year (Hitting mosquitoes with all available tools | Dengue | CDC).

## Preliminary Conclusions and Actions

In July 2025, ASDOH issued a dengue health alert that included recommendations for preventing mosquito bites while traveling and monitoring symptoms after returning. Because diagnostic testing was available on the island during this outbreak, the first dengue cases were confirmed within days rather than weeks, as was the case during previous outbreaks, when all samples had to be transported to Hawaii for dengue testing. In August 2025, CDC staff members provided dengue clinical management training to local health care professionals. Administrators updated the hospital’s surge plan to reallocate personnel and clinical space as needed to accommodate increased numbers of dengue cases. The American Samoa Environmental Protection Agency simultaneously led community cleanup campaigns to reduce *Aedes* species mosquito breeding sites and populations, resulting in the transport of approximately 250 large loads of household waste to the municipal landfill. ASDOH sprayed residual (long-lasting) insecticide at 52 day care centers, 33 public schools, 13 private schools, 11 government offices, six community health centers, five churches, one private business, and the hospital. Indoor residual spraying and outdoor residual spraying were offered to households of patients with dengue within 1–2 days of dengue diagnosis and to neighbors living within 164 ft (50 m) of affected residences.^§§^ As of June 15, 2026, among 740 households that could be reached by telephone, 716 (97%) had received outdoor residual spraying, and 297 (40%) had received indoor residual spraying. Beginning in March 2026, spatial emanators,^¶¶^ devices that passively emit a fast-acting insecticide called transfluthrin, were offered to households that declined indoor residual spraying; 422 spatial emanators were distributed to 77 households (an average of 5.5 per household), and 331 were installed in schools.

This is the first dengue outbreak for which American Samoa used on-island laboratory diagnostic testing to strengthen case identification. On-island laboratory diagnostic testing can be considered as an effective strategy to confirm DENV infections and guide rapid deployment of local vector control tools in future dengue outbreaks. Dengue surveillance and timely case reporting can guide outbreak response by facilitating the development and implementation of vector control tools and expediting clinical training when DENV transmission is identified.

## Footnotes

* Including nausea or vomiting, rash, headache, eye pain, joint pain, muscle aches, leukopenia, thrombocytopenia, abdominal pain or tenderness, extravascular fluid accumulation, mucosal bleeding, liver enlargement >2 cm, or increasing hematocrit level (CSTE case definition).

† InBios DENV Detect NS1 ELISA Kit; InBios Dengue Detect IgM Capture ELISA kit; and BioFire Defense Global Fever Panel

§ Detection of DENV and serotype identification was conducted using a CDC-developed assay.

¶ 45 C.F.R. part 46, 21 C.F.R. part 56; 42 U.S.C. Sect. 241(d); 5 U.S.C. Sect. 552a; 44 U.S.C. Sect. 3501 et seq.

** Population numbers are based on data from the U. S. Census Bureau | 2020 Island Areas Censuses: American Samoa.

†† The Lyndon B. Johnson Hospital is the only hospital in American Samoa and was under construction during the majority of the outbreak. Although the official listed capacity of the hospital is 150 beds, according to hospital administrators, the actual capacity was approximately 110 beds during the outbreak.

§§ Insecticides used in vector control activities included indoor residual spraying with deltamethrin (Suspend Polyzon: 5%) and bifenthrin (Talstar Professional and Bifen IT: 7.9%) and outdoor residual spraying with deltamethrin; bifenthrin combined with pyriproxyfen (Archer: 1.3%); or lambda-cyhalothrin, novaluron and pyriproxyfen (Proflex: 3.88%, 1.3%, and 1.3%, respectively). Aquatic habitats were treated with *Bacillus thuringiensis *subspecies* israelensis* (Mosquito Bits and briquettes) and spinosad (Natular Direct Tablet).

¶¶ Spatial emanators are devices that slowly release a fast-acting insecticide into the air, without using heat or electricity, that reduces mosquitoes’ ability to bite. The spatial emanator used during the outbreak contained transfluthrin and had a stated use duration of 1 year (Hitting mosquitoes with all available tools | Dengue | CDC).
